# Titanium Dioxide Nanoparticle-Driven Metabolic and Molecular Reprogramming in Cyanobacteria

**DOI:** 10.3390/molecules31172983

**Published:** 2026-08-26

**Authors:** Shyama Malika Malwalage, Mst Sayadujjhara, Viji Sitther

**Affiliations:** 1 Bioenvironmental Sciences Program, Morgan State University, Baltimore, MD 21251, USA; 2 Department of Biology, School of Computer, Mathematical and Natural Sciences, Morgan State University, Baltimore, MD 21251, USA

**Keywords:** cyanobacteria, photocatalytic–biological coupling, photosynthetic enhancement, reactive oxygen species, TiO_2_ nanoparticles

## Abstract

Cyanobacteria are promising platforms for bioenergy, carbon sequestration, and bioproduct synthesis, but their photosynthetic efficiency is limited by suboptimal light utilization, electron transport constraints, and environmental stress. Titanium dioxide nanoparticles (n-TiO_2_) have emerged as powerful photocatalytic materials that can enhance light absorption, modulate electron transport, and influence the redox balance in biological systems. This review advances the concept of photocatalytic-biological coupling, in which n-TiO_2_ functions as artificial light amplifiers that augment cyanobacterial photosynthesis. Current evidence on the physicochemical properties of n-TiO_2_, their interactions with cyanobacterial cells, and their effects on photosystems, electron transport chains, and downstream metabolic processes is examined. Particular emphasis is placed on the integration of photophysical and biological mechanisms, including reactive oxygen species (ROS)-mediated signaling, proton motive force (PMF) enhancement, and adenosine triphosphate (ATP) synthesis. Emerging approaches, including nano–bio interface engineering, environmental biotechnology applications, and artificial intelligence-guided optimization, are highlighted. By bridging photophysics, cellular bioenergetics, and computational design within a unified mechanistic framework, this review establishes the scientific foundation needed to translate photocatalytic–biological coupling into scalable and biotechnologically deployable nano-enabled photosynthetic systems.

## 1. Introduction

The accelerating global transition toward carbon-neutral energy systems and sustainable bioeconomy models has generated an urgent need for biological platforms capable of converting solar energy into high-value chemical products with minimal environmental footprint. Responsible for a substantial fraction of global primary productivity and oxygen evolution, cyanobacteria drive ecosystem function across marine and freshwater environments while simultaneously fixing atmospheric carbon dioxide (CO_2_) through highly coordinated photosynthetic machinery [1]. Beyond their ecological roles, cyanobacteria have demonstrated substantial biotechnological versatility, converting sunlight and CO_2_ into commercially relevant products including biofuels, bioplastics, phycobiliproteins, and pharmaceutical compounds [2,3]. These microorganisms can theoretically achieve solar-to-biomass conversion efficiencies of approximately 8–10%, which are substantially higher than those of conventional terrestrial bioenergy crops [4,5]. The capacity of cyanobacteria to grow on non-arable land using non-potable water, coupled with their amenability to genetic modification, has further reinforced their candidacy as next-generation cell factories for negative-emission biotechnologies. Nevertheless, the practical deployment of cyanobacterial production systems continues to be constrained by a fundamental bottleneck: the photosynthetic efficiency achieved under applied conditions remains far below the theoretical maximum, limiting volumetric productivity and economic competitiveness relative to heterotrophic microbial platforms.

Cyanobacteria possess a highly organized and functional photosynthetic apparatus. However, this system is inherently vulnerable to several efficiency-limiting mechanisms that become increasingly significant under large-scale cultivation conditions. One major constraint is photoinhibition, arising from high-irradiance damage to the D1 reaction center protein of photosystem II (PSII), which activates non-photochemical quenching (NPQ) that diverts absorbed photon energy away from photochemistry as protective heat dissipation [6]. Additional limitations arise from spectral restrictions associated with phycobilisome antenna complexes composed of phycocyanin, allophycocyanin, and phycoerythrin, which absorb light only within specific wavelength ranges, leaving substantial portions of the solar spectrum underutilized [7]. Under blue-light conditions, these constraints become particularly significant because inefficient energy transfer to PSII disrupts photosystem balance and reduces oxygen evolution and carbon fixation efficiency [8]. At the bioenergetic level, saturation of the plastoquinone pool generates bottlenecks in the electron transport chain (ETC), reducing the proton motive force available to drive F_0_F_1_-ATP synthase and limiting the ATP and NADPH supply required for the Calvin–Benson cycle [6]. Environmental stressors including fluctuating temperature, salinity, and UV radiation further dysregulate cellular redox homeostasis, compromising photosynthetic membrane integrity and metabolic flux [9]. Collectively, these intrinsic and extrinsic constraints present a major engineering challenge and overcoming them is central to unlocking the full biotechnological potential of cyanobacterial production systems.

Recent advances in nanotechnology have introduced a new class of interventions with the capacity to directly address these photosynthetic limitations at the biophysical and biochemical levels. Among the diverse array of engineered nanomaterials, titanium dioxide nanoparticles (n-TiO_2_) have attracted considerable scientific attention owing to their strong photocatalytic activity, UV-responsive wide bandgap optical properties, remarkable chemical stability, and relatively favorable safety profile at moderate concentrations [10,11]. This profile distinguishes n-TiO_2_ from other widely investigated semiconductor photocatalysts considered for biological applications. Graphitic carbon nitride (g-C_3_N_4_) and cadmium sulfide (CdS) have comparatively narrower band gaps (~2.7 and ~2.4 eV, respectively, versus ~3.0–3.2 eV for n-TiO_2_), providing greater responsiveness to visible light [12,13]. However, these materials also present limitations relevant to their use in biological systems. g-C_3_N_4_ can exhibit rapid recombination of photogenerated electron–hole pairs and relatively poor charge transport, which can limit photocatalytic efficiency despite its favorable visible-light absorption [12,13]. In contrast, CdS is susceptible to photocorrosion under prolonged illumination, with the potential release of Cd^2+^ ions posing additional concerns for biological applications [13]. Although limited by its wider band gap and predominantly UV-driven photoactivity, n-TiO_2_ offers high chemical and photostability. These characteristics, together with its well-established photocatalytic properties, make n-TiO_2_ particularly suitable for controlled interactions with living photosynthetic systems. Thus, while g-C_3_N_4_ and CdS offer advantages in visible-light utilization, n-TiO_2_ provides a distinct combination of stability and established nano–bio compatibility that is relevant to cyanobacterial applications.

Conventionally deployed in environmental remediation, photovoltaics, and antimicrobial applications, n-TiO_2_ is now being actively repositioned as functional modulators of biological photosynthesis. Titanium-containing nanoparticles have demonstrated consistent capacity to enhance chlorophyll synthesis, increase photosynthetic gas exchange parameters, and promote biomass accumulation across diverse photosynthetic organisms including microalgae and crop plants, with effects that are concentration-dependent, crystal phase-specific, and governed by surface-to-volume interactions with cellular components [14]. In cyanobacterial systems specifically, n-TiO_2_ exposure has been linked to enhanced PSII activity, increased phycobiliprotein pigment accumulation, elevated intracellular ATP content, and modulation of ROS levels through mechanisms that remain incompletely characterized at the molecular level [15]. These observations establish a compelling but mechanistically underexplored foundation for employing n-TiO_2_ as an active participant in cyanobacterial photosynthetic enhancement rather than as passive additives.

Despite the growing body of literature reporting n-TiO_2_ effects on photosynthetic microorganisms, significant knowledge gaps that limit rational system design and translational application remain. Most existing studies have been performed under idealized monoculture conditions using a narrow range of nanoparticle concentrations, with limited mechanistic integration across the photophysical, bioenergetic, and molecular levels of biological response. The absence of a unifying conceptual framework has further constrained the interpretability and comparability of findings across research groups and organisms. This review addresses these gaps by introducing and elaborating the concept of photocatalytic–biological coupling. This mechanistic framework reconceptualizes n-TiO_2_ not as an external chemical additive but as a functional artificial light amplifier that integrates with cyanobacterial photosystems to enhance photon capture, facilitate electron transport, and augment ATP synthesis. We critically synthesize current evidence on n-TiO_2_ physicochemical properties, nano–bio interface dynamics, photosystem interactions, ROS-mediated signaling, and bioenergetic outcomes, while further examining emerging opportunities in AI-guided structural optimization and environmental biotechnology applications. By providing this integrated mechanistic account, this review establishes a scientific foundation for the rational development of nano-enabled cyanobacterial systems with enhanced photosynthetic efficiency, improved bioproduct yields, and greater ecological sustainability at industrially relevant scales.

## 2. Cyanobacterial Photosynthesis and Its Inherent Limitations

Cyanobacterial photosynthesis operates through a highly interconnected bioenergetic network in which photosynthetic and respiratory processes share components within the thylakoid membrane system, enabling rapid adaptation to dynamic environmental conditions. Unlike higher plants, cyanobacteria possess integrated electron transport pathways that provide substantial metabolic flexibility and support survival across fluctuating light and nutrient environments [9]. This organization facilitates efficient coordination between light harvesting, electron transport, and cellular energy generation while simultaneously linking photosynthetic performance to intracellular redox regulation. Such functional integration allows cyanobacteria to sustain growth under variable conditions but also introduces vulnerabilities that can limit overall photosynthetic efficiency [16].

Despite this highly coordinated organization, cyanobacterial photosynthesis operates well below its theoretical maximum efficiency owing to a combination of intrinsic biochemical constraints and environmental pressures. As introduced above, spectral restrictions of phycobilisomes and photoinhibition of PSII represent two foundational and interconnected limitations, with the former leaving substantial portions of the solar spectrum unexploited and the latter diverting excitation energy away from photochemistry through NPQ activation when irradiance exceeds electron transport capacity [9,10]. ROS generated under such conditions further suppress D1 protein synthesis, impair PSII phosphorylation, and compound the rate of net photoinactivation [17]. Importantly, the efficiency of excitation energy transfer within phycobilisomes is constrained by the sparse chromophore arrangement and the Förster-type resonance energy transfer mechanism, which becomes particularly inefficient under blue-light illumination where photosystem I and II excitation becomes imbalanced, reducing oxygen evolution and overall quantum yield [9]. To mitigate progressive photodamage, cells engage photoprotective energy dissipation pathways including state transitions and orange carotenoid protein-mediated quenching; however, these protective mechanisms necessarily reduce the fraction of absorbed photons that contribute to productive carbon fixation, establishing an inherent trade-off between cellular protection and photosynthetic productivity that remains one of the central challenges in optimizing cyanobacterial systems for biotechnological deployment [18].

**Figure 1:**
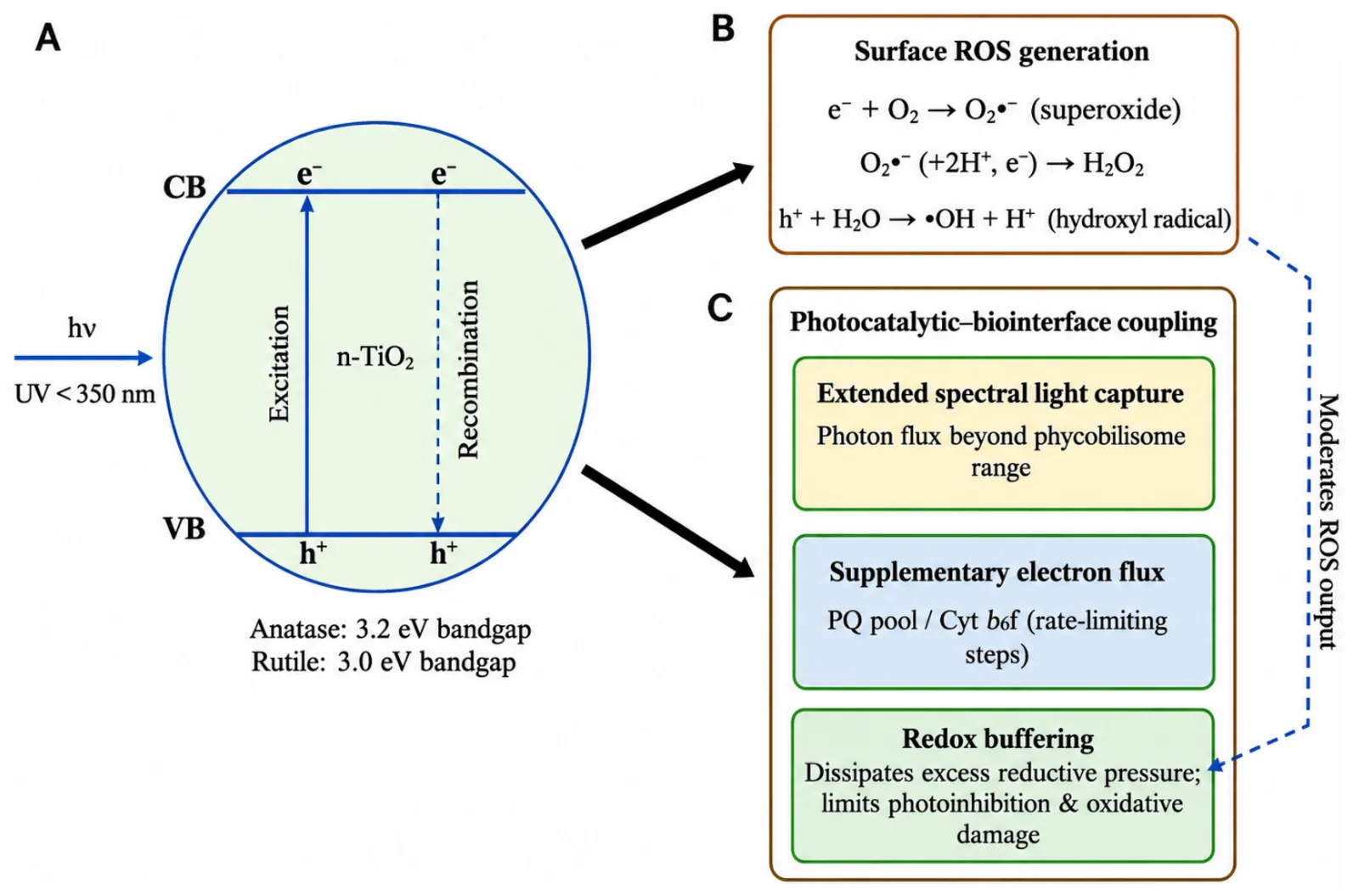
Photocatalytic activation of n-TiO_2_ and its coupling to cyanobacterial photosynthesis. (**A**) UV excitation generates electron–hole pairs (e^−^/h^+^); excitation competes with recombination. (**B**) Surface reactions generate ROS: e^−^ reduces O_2_ to superoxide and H_2_O_2_, while h^+^ oxidizes water to hydroxyl radical. (**C**) Charge carriers and ROS extend spectral light capture, supplement electron flux to the PQ pool/Cyt b_6_f, and drive redox buffering, which in turn moderates ROS output.

These inefficiencies are further compounded at the level of electron transport, where overreduction of the shared plastoquinone pool creates kinetic bottlenecks that restrict electron flux, attenuate proton gradient formation across the thylakoid membrane, and constrain the ATP and NADPH synthesis required for carbon fixation and biosynthetic metabolism [19]. Unlike higher plants, cyanobacteria share their plastoquinone pool between photosynthetic and respiratory electron transport, meaning that respiratory activity can directly compete with photosynthetic electron flow under fluctuating light conditions, further limiting the efficiency of linear electron transport and ATP production [9]. Under conditions of environmental stress, cyanobacteria activate cyclic electron flow and alternative electron transport pathways to preserve photosystem integrity and re-establish redox balance, yet these protective responses divert energy resources away from biomass accumulation and productive metabolic output [6]. Salinity, temperature fluctuations, and ultraviolet radiation exacerbate these dynamics by simultaneously impairing PSII electron transfer, suppressing D1 protein turnover, elevating intracellular ROS, and disrupting thylakoid membrane integrity in ways that compound photoinhibitory damage [20,21,22]. Collectively, these converging constraints across light harvesting, electron transport, and energy transduction define precise and interconnected physiological targets for intervention. Through photocatalytic charge generation, n-TiO_2_ can extend light absorption beyond the narrow range of phycobilisomes, facilitate electron transfer kinetics at rate-limiting steps in the transport chain, and modulate intracellular redox balance by supporting dissipation of excess reductive pressure that would otherwise drive photoinhibition and oxidative damage [23], positioning photocatalytic–biological coupling as a multifunctional strategy (Figure 1) targeting the spectral, kinetic, and redox limitations that constrain cyanobacterial photosynthetic efficiency.

## 3. Physicochemical Properties of Titanium Dioxide Nanoparticles: Determinants of Photocatalytic Activity

The photocatalytic and biological activity of n-TiO_2_ is fundamentally governed by their intrinsic physicochemical properties, which determine the efficiency of charge carrier generation, the yield and speciation of reactive oxygen species, and the nature of interactions with photosynthetic machinery. These material properties are established at the point of synthesis but can evolve under experimental conditions, making their systematic characterization under biologically relevant conditions an essential prerequisite for producing reproducible and mechanistically interpretable findings across biological systems [24,25].

### 3.1. Crystal Phase Composition

Among the intrinsic physicochemical determinants of n-TiO_2_ photocatalytic performance, crystal phase composition most directly controls charge carrier recombination kinetics and ROS generation efficiency through its influence on electronic band structure. As illustrated in Figure 1, photocatalytic activity in n-TiO_2_ is initiated when UV irradiation promotes electrons (e^−^) from the valence band to the conduction band, generating electron–hole pairs (e^−^/h^+^) whose fate, whether productive surface redox chemistry or unproductive recombination, is critically determined by crystal phase composition [24,26]. Anatase consistently demonstrates superior photocatalytic activity under UV irradiation, attributable to its wider bandgap of approximately 3.2 eV compared to 3.0 eV for rutile, a conduction band position more negative than the O_2_/O_2_^−^ redox potential that facilitates superoxide generation, and a substantially lower electron–hole recombination rate arising from its indirect bandgap nature and longer charge carrier lifetimes [24,27]. As shown in Figure 1, this slower recombination permits a greater fraction of photogenerated charge carriers to reach the nanoparticle surface and participate in productive redox reactions, directly translating into higher ROS yields and greater capacity for photocatalytic electron transfer to cyanobacterial photosynthetic components [26,28].

Rutile, while less photocatalytically active in isolation due to its direct bandgap and correspondingly faster recombination rate (Figure 1), exhibits stronger visible light absorption owing to its narrower bandgap of 3.0 eV, and within mixed-phase systems functions as a visible-light antenna that extends photoactivity beyond the UV range [29]. Mixed-phase materials, most prominently the widely studied P25 formulation (approximately 75% anatase, 25% rutile), exhibit photocatalytic activity substantially exceeding either pure phase alone; electron paramagnetic resonance spectroscopy has demonstrated that charges generated on rutile under visible light are stabilized through rapid electron transfer to lower-energy anatase lattice trapping sites, creating catalytic hot spots at phase boundaries that suppress recombination and prolong the lifetime of reactive surface species [29,30]. Brookite, the least studied polymorph, possesses shallow electron traps that extend hole lifetime relative to rutile, conferring intermediate photocatalytic activity; however, its charge carrier dynamics and specific role in cyanobacterial photosynthetic responses remain largely uncharacterized [31,32]. Crystal phase composition should therefore be confirmed by X-ray diffraction with Rietveld refinement to quantify phase ratios, and X-ray photoelectron spectroscopy to characterize surface oxidation states, as these data are essential for mechanistic attribution of observed biological effects to the specific charge separation and recombination dynamics depicted in Figure 1 [24].

### 3.2. Particle Size, Morphology, and Surface Area

Particle size and morphology are interconnected determinants of the surface-to-volume ratio, which governs the density of photocatalytically active surface sites and the probability of interaction with cyanobacterial cell surfaces and photosynthetic components. Smaller n-TiO_2_ particles present proportionally greater surface area per unit mass, increasing the density of surface hydroxyl groups available for ROS generation [24]. Morphological variation, encompassing spherical particles, nanorods, nanotubes, and nanosheets, introduces additional complexity by altering the distribution of exposed crystal facets, with anatase {001} facets demonstrating substantially higher photocatalytic reactivity than thermodynamically stable {101} facets due to differences in surface atomic geometry and hydroxylation density [33]. Surface area, quantified by N_2_ physisorption analysis, provides an integrated measure of the reactive interface available for photocatalysis and should be reported alongside particle size measurements to enable cross-study comparability [24]. Transmission electron microscopy and scanning electron microscopy provide direct visualization of primary particle size and morphology and should be reported alongside dynamic light scattering measurements of hydrodynamic diameter, as the discrepancy between these values directly quantifies the degree of aggregation occurring under experimental conditions, a critical parameter that substantially alters the effective biological dose delivered to cyanobacterial cells [25] (Table 1).

### 3.3. Bandgap Properties and Light Responsiveness

A fundamental but frequently underappreciated constraint on the biological efficacy of n-TiO_2_ in cyanobacterial systems is the spectral mismatch between the UV-confined absorption range of pristine anatase and the visible and red-enriched illumination conditions characteristic of photobioreactor cultivation, a consideration that critically affects the interpretation of experimental outcomes across studies [39,40]. Under UV-inclusive white fluorescent illumination, n-TiO_2_ photocatalysis proceeds efficiently, generating ROS that interact with cellular components; under red-filtered or narrow-band visible illumination, the same nanoparticles may function primarily through physical surface interactions rather than photocatalytic mechanisms, producing qualitatively distinct biological responses [40,41]. Surface modification strategies including nitrogen doping, carbon coating, and metal ion incorporation have been employed to extend the effective absorption range of n-TiO_2_ into the visible spectrum, with nitrogen-doped variants demonstrating photocatalytic activation under visible light below 500 nm [39,42]. Diffuse reflectance UV–visible spectroscopy with Tauc plot analysis enables accurate determination of the optical bandgap and identification of sub-bandgap absorption features introduced by doping or surface modification, providing data essential for predicting photocatalytic behavior under the specific illumination conditions of a given biological experiment [42] (Table 1).

Pure anatase n-TiO_2_ is intrinsically limited by its wide band gap (approximately 3.0 to 3.2 eV), which confines photoactivation to the UV fraction of sunlight and constrains its use in outdoor or field-deployed systems. Non-metal doping narrows this band gap and red-shifts absorption into the visible range. Nitrogen doping introduces N 2p states above the valence band [43], while carbon doping enhances charge separation through similar mid-gap states [37]. In cyanobacteria specifically, nitrogen-doped and carbon/nitrogen co-doped TiO_2_ formulations have shown markedly faster visible-light photocatalytic activity against the cyanotoxin microcystin-LR than undoped TiO_2_ [44]. At the physiological level, N-doped anatase produced a distinctly different response in *Arthrospira platensis* than pure anatase, eliciting an adaptive antioxidant polyphenol response rather than the 74.1% growth inhibition seen with undoped anatase [45], consistent with the concentration-dependent divergence already summarized in Table 2. This indicates that doping shifts not only the light regime of activation but also the threshold separating adaptive from inhibitory responses. Direct measurements of PSII efficiency, electron transport, and ATP synthase activity following exposure to doped n-TiO_2_ remain limited in cyanobacteria, despite evidence that pristine TiO_2_ can affect these photosynthetic and metabolic processes.

### 3.4. Surface Hydroxylation and ROS Generation Capacity

Surface hydroxyl groups (Ti-OH) serve as the primary photocatalytic sites where photogenerated holes and molecular oxygen undergo redox reactions to yield ROS, and their density directly governs both the magnitude and speciation of the photocatalytic oxidative output under a given illumination condition [28]. ROS regulation is an active area of photocatalysis research, with recent work demonstrating that structural engineering of alternative photocatalysts, such as carbon nitride, can selectively favor specific ROS species over others [51]. Single-atom catalyst systems have similarly shown that water availability at catalytic sites critically governs ROS generation pathways, underscoring principles that may extend to nanoparticle–biological interfaces generally [52]. The identity of ROS species generated, including hydroxyl radicals, superoxide, and hydrogen peroxide, reflects the interplay between surface hydroxylation density, crystal phase, light intensity, and the availability of electron acceptors in the surrounding medium, with each species conferring mechanistically distinct biological effects on cyanobacterial photosynthetic components [28,53]. Hydroxyl radicals, generated through direct hole transfer to surface-bound Ti-OH groups, are highly reactive and spatially confined oxidants that damage membrane lipids and PSII reaction center proteins within nanometer-scale proximity of the nanoparticle surface. Superoxide and hydrogen peroxide, produced via electron transfer to dissolved oxygen, are comparatively longer-lived and diffusible, enabling ROS-mediated signaling at distances beyond the immediate nano-bio contact zone and at sub-toxic concentrations relevant to oxidative eustress [28,54]. Species-resolved measurements directly confirm that crystal phase governs which ROS n-TiO_2_ predominantly generates and how far those species travel from the particle surface. Using terephthalic acid fluorescence probing with single-molecule detection, Kim et al. [55] showed that hydroxyl radicals formed on anatase desorb and diffuse freely into bulk solution, while hydroxyl radicals formed on rutile stay adsorbed at the surface and are largely unavailable to react with substrates in solution, a mobility difference proposed as the main reason anatase outperforms rutile in reactions involving dissolved substrate. Coumarin-based probes that distinguish surface-bound from bulk-diffused hydroxyl radicals provided further evidence for this difference and linked it to distinct surface reaction pathways: on anatase, trapped holes directly generate adsorbed hydroxyl radicals, whereas on rutile, hydroxyl radical formation proceeds through a surface Ti-peroxo intermediate. Consistent with these differences, H_2_O_2_ addition increases hydroxyl radical production on rutile but decreases it on anatase [28]. Hirakawa et al. [56] quantified this phase dependence across both major ROS using chemiluminescence and fluorescence probes, finding hydroxyl radical formation consistently higher on anatase while rutile more effectively stabilized superoxide, indicating that phase controls not just total ROS yield but the partitioning between hydroxyl radical and superoxide pathways. The ability of freely diffusing hydroxyl radicals and longer-lived superoxide and H_2_O_2_ species to reach cytoplasmic and membrane-distal targets, unlike surface-confined oxidants, highlights the importance of phase-dependent ROS speciation, rather than ROS yield alone, in interpreting biological outcomes in n-TiO_2_–cyanobacteria systems. Surface hydroxyl density, quantifiable by thermogravimetric analysis or diffuse reflectance infrared Fourier transform spectroscopy, is therefore a mechanistically critical parameter whose systematic characterization is essential for predicting the ROS speciation profile generated by a given n-TiO_2_ formulation and for distinguishing photocatalytic from physical interaction modes under the illumination conditions of a given biological experiment [24,28,57] (Table 1).

### 3.5. Systematic Characterization Framework

Reproducible and mechanistically interpretable findings across research groups require systematic and multiparametric characterization of n-TiO_2_ under biologically relevant conditions. Individual physicochemical parameters cannot be evaluated in isolation, as crystal phase, particle size, surface area, agglomeration state, bandgap, and surface hydroxylation collectively and interdependently govern both photocatalytic performance and biological interaction outcomes. Table 1 presents a recommended characterization workflow that maps each analytical technique to the specific physicochemical property it quantifies and its direct biological relevance in cyanobacterial photosynthesis research. Reporting this complete parameter set as a minimum standard would substantially improve cross-study comparability, enable mechanistic attribution of observed biological effects to specific nanoparticle properties, and support the rational design of n-TiO_2_ supplementation strategies for photosynthetic enhancement applications.

## 4. Mechanisms of Nano–Bio Interface Formation and Cellular Interactions Between n-TiO_2_ and Cyanobacteria

**Figure 2:**
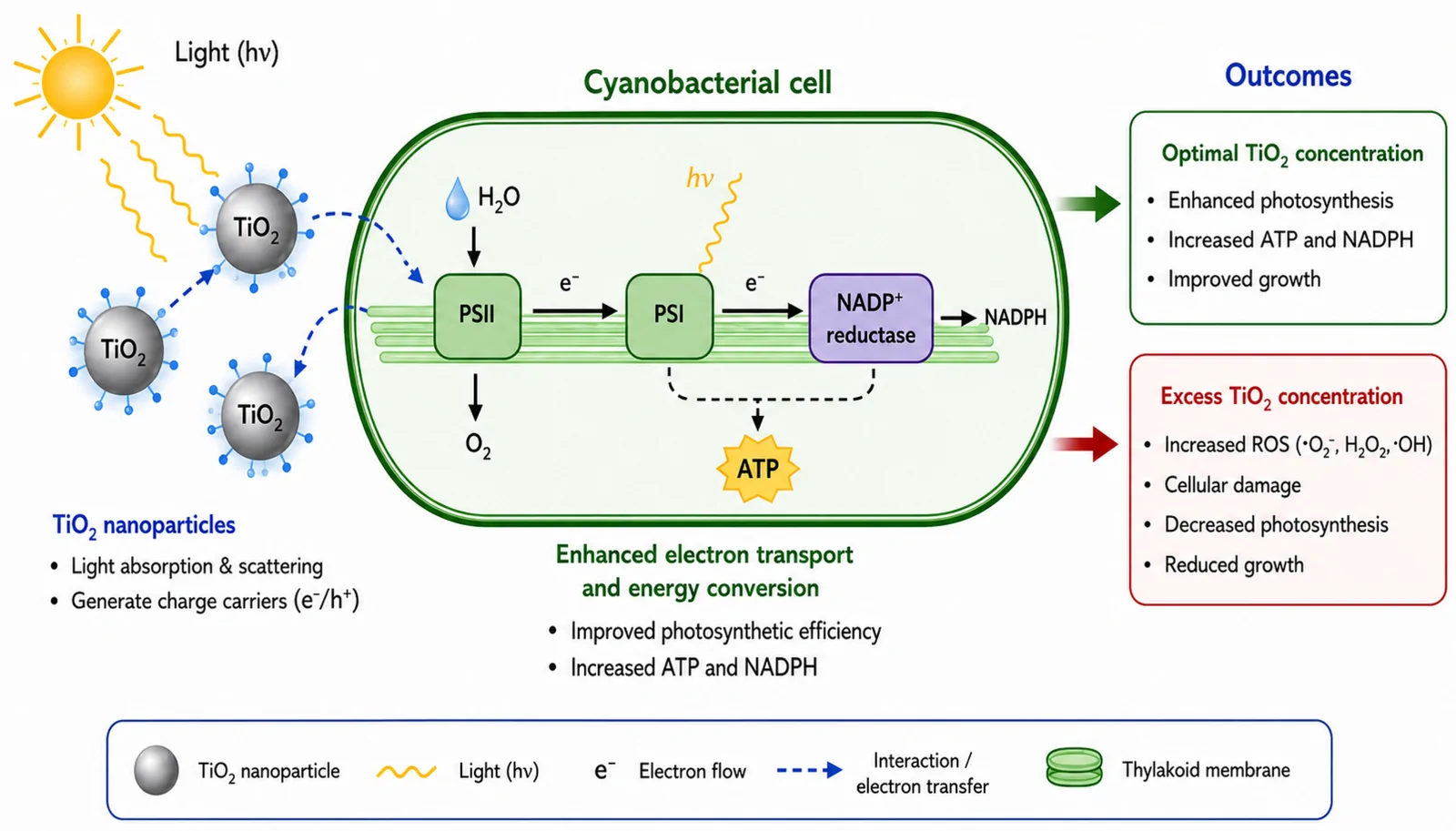
Mechanistic model of photocatalytic–biological coupling in cyanobacteria mediated by Titanium dioxide (TiO_2_) nanoparticles.

The biological identity of n-TiO_2_ is substantially transformed before cellular contact occurs. Upon introduction into culture media, nanoparticle surfaces rapidly adsorb proteins, polysaccharides, and lipids to form a structurally layered biomolecular corona comprising an inner hard corona of tightly and irreversibly bound molecules and an outer soft corona of loosely associated, rapidly exchanging species [58]. The ionic strength conditions characteristic of cyanobacterial mineral salt media further stabilizes hard corona formation by compressing the electrical double layer and promoting hydrophobic protein–surface contacts. This drives a transition from transient to persistent corona configurations that fundamentally redefine nanoparticle surface charge, aggregation kinetics, and cellular recognition relative to measurements conducted in simple aqueous suspension [35,59] (Figure 2).

A critical but underappreciated dimension of the nano–bio interface in cyanobacterial systems is the interception of n-TiO_2_ by extracellular polymeric substances prior to cell wall contact. Cyanobacterial EPS are heterogeneous assemblages of proteins, polysaccharides, and humic compounds bearing amino, carboxyl, and phenolic functional groups that envelop nanoparticles to form eco-corona layers or crosslink them into bridged aggregates, profoundly altering colloidal stability, effective particle size, and ROS-generating surface chemistry [36,60]. Notably, the crystal phase and exposed facets of n-TiO_2_ govern the selectivity and extent of EPS adsorption. Consequently, the same nanoparticle formulation can produce divergent eco-corona compositions and aggregation states depending on strain-specific EPS secretion profiles, introducing biological variability that single-parameter physicochemical measurements cannot resolve [36,61].

Nanoparticles that successfully traverse the EPS matrix and corona layers engage the cyanobacterial cell wall and thylakoid membrane system, where the concentration regime becomes the decisive determinant of biological outcome. Direct ultrastructural evidence provides mechanistic insight into the penetration of n-TiO_2_ across the cyanobacterial cell envelope. In the cyanobacterium *Anabaena variabilis,* extracellular biomolecules, particularly extracellular DNA, mediated the disaggregation of n-TiO_2_ aggregates at the cell surface, increasing the number of individual particles small enough to cross the peptidoglycan layer and outer membrane [62]. The resulting increase in dispersed nanoparticles enhanced their ability to cross the peptidoglycan layer and outer membrane, facilitating cellular internalization. Internalization of n-TiO_2_ was associated with several ultrastructural alterations, including the formation of a membrane-associated mucilage layer, an increased number of cytoplasmic crystalline inclusions, expansion of the intra-thylakoid space, disruption of the plasma membrane, and elevated intracellular ROS levels. These findings indicate that particle size and aggregation state, not exposure concentration alone, determine whether n-TiO_2_ remains surface-adsorbed or becomes internalized, and that the thylakoid membrane system is a direct target of internalized particles rather than a downstream effect of surface oxidative stress. At low to moderate exposures, n-TiO_2_ association with thylakoid membranes modulates membrane fluidity and proton translocation efficiency in ways consistent with photosynthetic enhancement [63]. At excessive concentrations, physical intercalation into thylakoid membranes disrupts the lateral supramolecular organization of PSII and PSI complexes, impairing the spatially coordinated electron transport architecture on which efficient photosynthesis depends [63,64]. Strain-specific differences in EPS composition, cell wall architecture, and antioxidant capacity further modulate these outcomes at every interaction layer, producing divergent pigment fluorescence and ROS accumulation profiles across genetically distinct cyanobacterial backgrounds and underscoring the necessity of strain-resolved experimental designs in this field (Figure 2).

## 5. Photocatalytic–Biological Coupling Mechanism of n-TiO_2_ in Cyanobacteria

### 5.1. Enhancement of Light Capture

**Figure 3:**
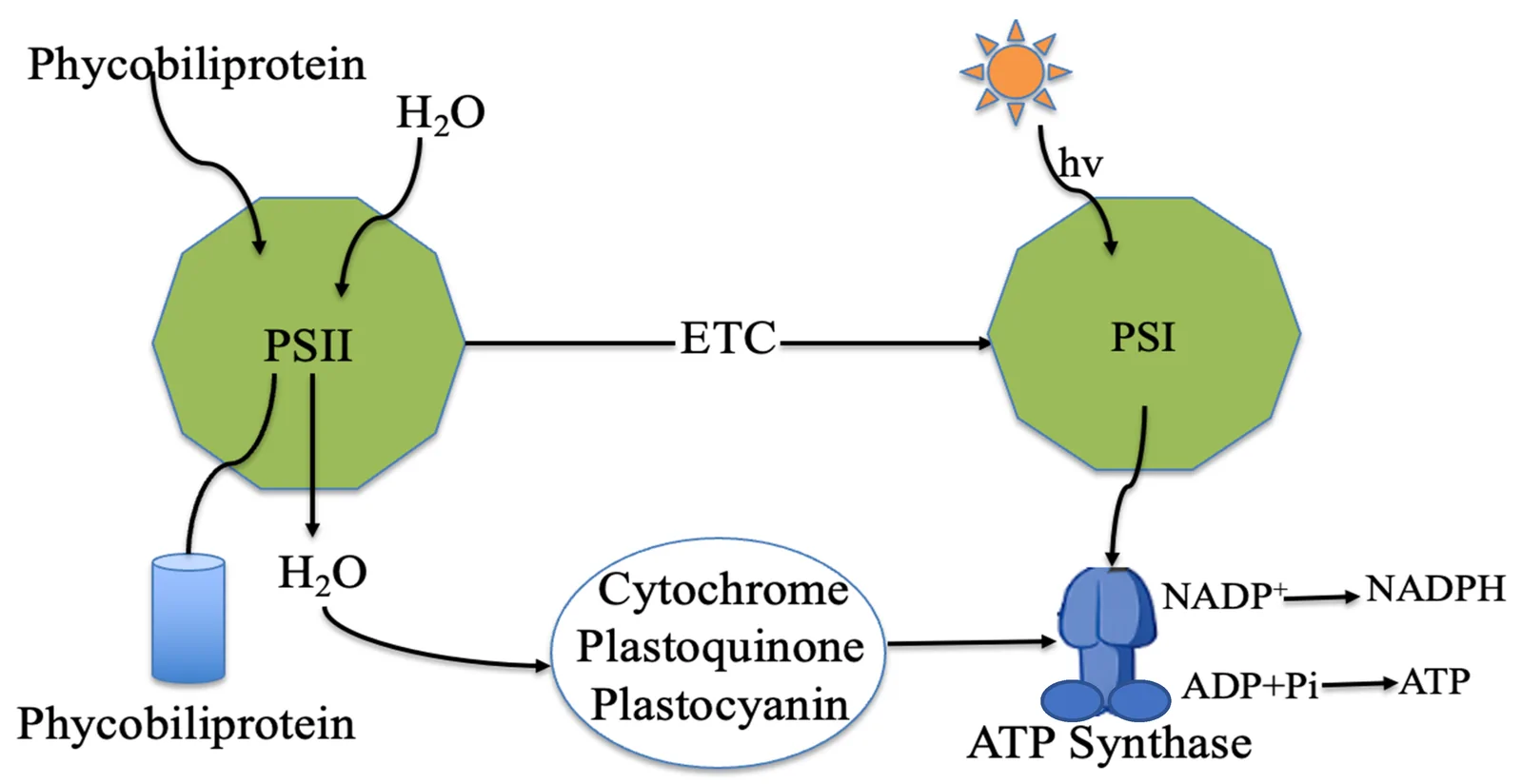
Schematic representation of cyanobacterial photosynthetic electron transport and energy conversion.

Among the most consequential interactions between n-TiO_2_ and cyanobacterial photosynthesis is the capacity of nanoparticles to augment photon availability at pigment complexes, directly addressing the spectral limitations inherent to phycobilisome-based light harvesting (Figure 3). Photoactivated n-TiO_2_ redistributes incident radiation spatially via light scattering and near-field optical enhancement, concentrating photons within the phycobilisome absorption range near the nanoparticle surface and thereby increasing local photon flux without spectral shift [65]. At optimized concentrations, this photon redistribution improves PSII excitation efficiency, increasing water oxidation rates and the release of electrons into the transport chain. In *Fremyella diplosiphon*, an optimal n-TiO_2_ concentration of 2 mg/L enhanced phycobiliprotein autofluorescence and PSII quantum efficiency [46], indicating that light harvesting and excitation energy transfer within the photosynthetic antenna may be important biological targets of photocatalytic–biological interactions in cyanobacterial systems. Evidence from plant chloroplast systems demonstrates that n-TiO_2_ exposure concurrently elevates RuBisCO and ATP synthase activity alongside increased NADPH production, indicating that light capture enhancement propagates through the full photosynthetic energy conversion cascade rather than being confined to the antenna level [66,67].

### 5.2. Modulation of Electron Transport

Photocatalytically generated charge carriers on the surface of photoactivated n-TiO_2_ interact directly with components of the photosynthetic electron transport chain, providing supplementary electron flux that reduces recombination losses at rate-limiting steps. Critically, n-TiO_2_-derived electrons enter the transport chain at the level of the plastoquinone pool and cytochrome b6f complex, the major proton-pumping and electron-coupling hub whose turnover rate determines the efficiency of PMF generation across the thylakoid membrane [6,65] (Figure 3). In *Synechocystis* sp. PCC 6803, n-TiO_2_ treatment produces a substantial increase in photocurrent generation, directly demonstrating nanoparticle capacity to amplify photosystem-driven electron transport beyond levels achievable by photosynthesis alone [23]. The resulting strengthening of PMF drives F_0_F_1_-ATP synthase at higher rates, increasing ATP output and expanding the metabolic capacity available for carbon fixation and biosynthesis. Collectively, nanoparticle-mediated improvements in electron transport efficiency have been associated with photosynthetic performance gains approaching 30% under optimized conditions, establishing electron transport modulation as the primary bioenergetic mechanism through which photocatalytic–biological coupling translates into measurable productivity enhancement [46].

### 5.3. Photocatalytic Reactive Oxygen Species Generation: From Oxidative Stress to Adaptive Signaling

The dual role of n-TiO_2_-generated ROS as both productive signaling agents and cytotoxic oxidants is inseparable from the evolutionary context of ROS biology in oxygenic phototrophs. Cyanobacteria generate ROS endogenously during normal photosynthesis, particularly when electron transport outpaces CO_2_ fixation, producing superoxide at PSI that superoxide dismutase converts to H_2_O_2_ for subsequent catalase-mediated detoxification [53]. This pre-existing redox signaling infrastructure, encompassing superoxide dismutase, catalase, peroxiredoxins, glutathione, and carotenoids, confers an intrinsic capacity to perceive and respond adaptively to low-level exogenous ROS, including those generated by n-TiO_2_ photocatalysis, rather than treating them exclusively as damage signals [68,69]. Within this evolutionary framework, photoactivated n-TiO_2_ generates electron–hole pairs whose surface reactions with H_2_O and dissolved O_2_ produce hydroxyl radicals, superoxide, and H_2_O_2_ in proportions governed by crystal phase, surface area, and light intensity [28,70].

At sub-toxic concentrations, photocatalytically generated H_2_O_2_ is proposed to operate within the oxidative eustress regime, potentially inducing reversible post-translational modifications of redox-sensitive cysteine residues, including sulfenylation, disulfide bond formation, and S-glutathionylation, that may function as molecular switches activating protective gene expression programs, upregulating antioxidant defenses [53,71]. Direct evidence for these specific modifications in response to n-TiO_2_-generated ROS is currently limited in cyanobacteria; this mechanistic framework draws largely from redox signaling studies in plant and non-photosynthetic systems and has not yet been directly demonstrated in these microorganisms. Importantly, this photocatalytic ROS production is attributable to the nanoparticles themselves rather than to dissolved Ti ions, and its magnitude varies substantially with nanoparticle formulation and the ionic composition of the culture medium, a distinction that is critical for translating laboratory findings to environmentally and biotechnologically realistic deployment conditions [28,37]. As n-TiO_2_ concentration increases beyond the adaptive threshold of the antioxidant network, the response transitions from productive eustress to oxidative distress, manifested as lipid peroxidation quantified by malondialdehyde accumulation, protein carbonylation, nucleic acid damage, thylakoid membrane disruption, and progressive loss of cell viability [53,63]. Precisely defining this concentration-dependent biphasic transition for each cyanobacterial strain, culture condition, and nanoparticle formulation therefore represents a non-negotiable prerequisite for any biotechnological deployment of n-TiO_2_-augmented photosynthetic systems.

## 6. Effects of n-TiO_2_ on Cyanobacterial Photosystems and Electron Transport

Empirical evidence from diverse experimental systems demonstrates concentration-dependent effects of n-TiO_2_ on cyanobacterial photosystem function. At optimally low concentrations, n-TiO_2_ stabilizes the structural integrity of PSII reaction center proteins D1 and D2 by attenuating oxidative damage, thereby sustaining electron generation from water splitting [28,65]. Enhanced electron flow through the ETC reduces NPQ, ensuring that a greater proportion of absorbed photon energy is directed toward photochemistry rather than dissipated as heat [15].

Molecular-level evidence for n-TiO_2_–ETC interactions from transcriptomic studies indicates that low concentrations of n-TiO_2_ upregulate the expression of genes encoding photosystem subunits, including psaA, psaB (PSI), psbA (D1 protein of PSII), and atpB (ATP synthase β-subunit) in microalgal systems [49]. Conversely, exposure to environmentally relevant concentrations of n-TiO_2_ can suppress expression of these same genes, indicating that photosynthetic enhancement and inhibition occupy distinct concentration regimes [49] (Table 2). In the specific context of *F. diplosiphon*, strain B481-SD (overexpressing the sterol desaturase gene) exhibited enhanced PSII activity and pigment autofluorescence under n-TiO_2_ treatment, whereas the wild-type strain B481-WT showed differential sensitivity, underscoring the importance of genetic background in mediating nanoparticle responses [46] (Table 2). This strain-specific difference is consistent with the documented membrane-level effect of sterol desaturase overexpression: B481-SD maintains a higher baseline ratio of unsaturated to saturated fatty acids than B481-WT, and this elevated membrane unsaturation has been associated with enhanced tolerance to nanoparticle-induced and other oxidative stressors in this strain [9]. Greater membrane fluidity may buffer thylakoid membrane integrity against n-TiO_2_-induced oxidative stress, plausibly preserving PSII function and ATP synthase activity at concentrations that begin to compromise the less fluid membrane of the wild type. At excessive concentrations, n-TiO_2_ induces a dysregulation of photosystem function through overproduction of ROS, leading to PSII core protein damage, plastoquinone pool oxidation, and impaired proton gradient formation [15,48]. These concentration-dependent biphasic effects demand precise calibration of n-TiO_2_ dosage relative to cell density, light intensity, and species-specific sensitivity.

The apparent discrepancy between studies reporting enhancement and inhibition at similar n-TiO_2_ concentrations reflects differences in particle characteristics and species sensitivity rather than a true contradiction. *F. diplosiphon* B481-SD showed enhancement at 2.0 mg L^−1^ using pure anatase particles (~21 nm), whereas *M. aeruginosa* and *Synechococcus cedrorum* showed no enhancement and an IC_25_ of 2.7 mg L^−1^ using P25, a larger (81.5 nm) anatase-rutile mixed-phase particle [47,50] (Table 2). As discussed in Section 3, mixed-phase P25 generates a distinct ROS profile from pure anatase, and larger crystallite size reduces surface reactivity and cellular uptake efficiency. Species- and strain-specific differences in EPS production and cell wall structure further modulate nanoparticle–cell interactions, so a concentration that is sub-inhibitory for one organism can exceed the tolerance threshold of another. These findings underscore that n-TiO_2_ concentration alone is an insufficient predictor of biological outcome; crystal phase, particle size, and species identity must be considered jointly when comparing studies.

## 7. Bioenergetic Outcomes: ATP Synthesis and Metabolic Enhancement

The downstream consequence of enhanced electron transport and strengthened PMF is elevated F_0_F_1_-ATP synthase activity, which represents the critical conversion point at which photocatalytic–biological coupling translates into measurable metabolic gain. In cyanobacteria and chloroplasts, ATP synthase plays a pivotal role as a photosynthetic membrane complex responsible for producing ATP from ADP and inorganic phosphate utilizing a proton motive force gradient induced by photosynthesis, and the structural and functional similarities between cyanobacterial and chloroplast ATP synthase suggest that the cyanobacterial form is the evolutionary precursor to the chloroplast complex [72]. The F_0_ complex converts the potential energy of the thylakoid membrane into rotary force by binding and dissociating with protons, while the F_1_ complex utilizes this rotary force to catalyze ATP synthesis, serving as the site where potential energy is ultimately converted into biologically usable chemical energy [72,73]. Critically, ATP synthase can degrade ATP when the PMF across the thylakoid membrane falls below a certain threshold, meaning that stable and sufficient PMF is an absolute prerequisite for net ATP synthesis rather than hydrolysis [72,74]. The conditions promoted by low to moderate n-TiO_2_ concentrations, namely strengthened PMF and attenuated oxidative stress at photosynthetic membranes, directly satisfy this prerequisite and thereby sustain productive ATP synthase operation [74].

Fluctuating light and dark periods, common in outdoor cultivation and densely mixed photobioreactors, raise a distinct question from the steady-state case above, and one with direct bearing on bioprocess design: whether n-TiO_2_ exposure could inadvertently promote reverse, hydrolytic ATP synthase activity under these conditions. In cyanobacteria specifically, this risk is constrained by a lineage-specific safeguard: unlike the chloroplast enzyme, whose γ subunit carries a redox-active dithiol/disulfide switch that shuts down hydrolysis in the dark, the cyanobacterial γ subunit lacks this Cys-based redox regulation entirely, and ATP hydrolysis is instead suppressed by an ADP-dependent inhibitory mechanism together with the small membrane protein ATPθ, whose levels rise under dark or low-energy conditions specifically to arrest wasteful ATP hydrolysis [75]. N-TiO_2_ photocatalysis is itself light-driven; its PMF-enhancing effect should therefore switch off along with illumination rather than persist into dark periods, and the nanoparticles are not expected to actively drive reverse-mode hydrolysis once the ATPθ -mediated brake engages. The more plausible risk for bioprocess design instead sits at the transition between states: abrupt shifts between illuminated and shaded conditions, as occur through self-shading in dense outdoor cultures, diel cycling, or mixing-induced excursions in and out of the photic zone, could create a brief window before PMF collapses and ATPθ-mediated inhibition is fully established, and at higher, distress-regime n-TiO_2_ concentrations, membrane oxidative damage could in principle interfere with this regulatory system. Whether n-TiO_2_ exposure measurably widens this transition window under realistic fluctuating-light cultivation has not been directly tested, and we identify it here as an open question relevant to scale-up, where diurnal cycling and self-shading are unavoidable.

Increased ATP availability propagates through the cyanobacterial metabolic network by directly fueling carbon fixation through the Calvin–Benson cycle and supporting anabolic processes including pigment biosynthesis, protein synthesis, and lipid metabolism. The addition of low-concentration TiO_2_ nanoparticles effectively promotes cyanobacterial growth through increased chlorophyll levels, resulting in enhanced photosynthetic efficiency and facilitated accumulation of stored biomolecules [76]. In *A. platensis*, n-TiO_2_ treatment has been documented to improve total protein and pigment yields through effects linked mechanistically to PSII activity [15], while in *F. diplosiphon*, strain-specific increases in phycobiliprotein accumulation under optimized n-TiO_2_ exposure reflect the downstream biosynthetic consequences of enhanced photosynthetic energy supply [46]. Enhanced NADPH generation, as a product of amplified PSI activity, further broadens this metabolic benefit by supplying the reducing equivalents required for biosynthetic reductive pathways. The convergence of elevated ATP supply and increased NADPH availability therefore represents the metabolic translation of photocatalytic–biological coupling, in which upstream improvements in light capture and electron transport propagate through the bioenergetic network to yield measurable gains in biomass productivity, pigment accumulation, and bioproduct output.

## 8. Omics-Level Responses of Cyanobacteria to n-TiO_2_ Exposure

### 8.1. Transcriptomic Reprogramming

The ultrastructural consequences of n-TiO_2_ internalization documented in Section 4, including thylakoid disruption, membrane-associated mucilage formation, and elevated intracellular ROS, raise questions on the molecular programs driving these changes. Transcriptomic analyses have provided a genome-wide view of the gene expression changes induced by n-TiO_2_ exposure in photosynthetic microorganisms, revealing coordinated activation of stress-protective pathways alongside modulation of photosynthetic capacity. Transcriptomic evidence across multiple n-TiO_2_-exposed biological systems documents consistent upregulation of oxidative stress response networks, including antioxidant enzymes, DNA repair machinery, and heat shock proteins, as conserved transcriptional signatures of nanoparticle-induced ROS accumulation [77]. At the level of photosynthetic gene regulation, exposure of Chlorella vulgaris to environmentally relevant n-TiO_2_ concentrations induced divergent transcriptional responses, with significant repression of rbcL at 1.1 to 4.4 µg/L indicating early inhibition of carbon fixation, and upregulation of psaA and psaD at 8.8 to 17.6 µg/L suggesting compensatory reinforcement of photosystem I activity [49] (Table 3). These concentration-dependent, gene-specific responses demonstrate that molecular endpoints reveal sublethal effects not detectable through growth measurements alone [49], indicating the importance of multi-gene transcriptional profiling for characterizing nanoparticle-photosystem interactions (Table 3).

### 8.2. Proteomic Alterations

Proteomic studies of cyanobacteria exposed to abiotic stress and nanomaterials have demonstrated coordinated reprogramming of photosynthetic, chaperone and metabolic protein networks that complement and extend transcriptional responses. Using TiO_2_ enrichment combined with LC-MS/MS-based global phosphoproteomic analysis, Yang et al. identified 245 proteins harboring 410 phosphorylation sites across 280 phosphopeptides in *Synechococcus* sp. PCC 7002, with characterized phosphoproteins involved in two-component signal transduction pathways and photosynthetic reactions [78]. The study established that post-translational modification of photosynthetic signaling proteins represents a key dimension of the cyanobacterial stress response that cannot be captured by transcriptomics alone. A systematic survey of published proteomic studies confirmed that photosynthesis-related proteins, including those involved in PSII core function and phycobiliprotein assembly, are among the most consistently differentially abundant proteins across multiple cyanobacterial species subjected to diverse abiotic stressors [79].

Under n-TiO_2_ exposure, the protein quality control network is prominently activated. Studies using n-TiO_2_ in plant systems have reported proteomic changes associated with upregulation of stress-responsive proteins, including members of the chaperone network [80]. Chaperone systems such as GroEL/GroES and DnaK are well-established components of the universal stress proteome in cyanobacteria [79]. Their increased abundance under nanoparticle-induced proteotoxic stress reflects the cellular investment required to preserve photosynthetic complex integrity through protein refolding. Metabolic enzymes involved in carbon fixation and antioxidant metabolism also exhibit concentration-dependent abundance changes that parallel transcriptomic patterns, confirming that nanoparticle responses are coordinated across multiple regulatory levels.

### 8.3. Metabolomic Responses

Metabolomic profiling provides a direct functional readout of the metabolic consequences of n-TiO_2_ exposure by quantifying the net outcome of transcriptional, translational, and enzymatic changes on cellular metabolite pools. Untargeted metabolomic analysis of *Chlamydomonas reinhardtii* exposed to n-TiO_2_ has revealed that exposure reprograms lipid and amino acid metabolism, suppresses protein biosynthesis, and perturbs central pathways including the TCA cycle, photosynthesis, and carbohydrate metabolism, leading to disruptions in energy balance and metabolic homeostasis [81]. Consistent with the oxidative distress regime described in Section 5.3, this membrane damage is detectable metabolomically as elevated malondialdehyde accumulation and increased membrane permeability [15,48], providing the chemical footprint of a transition already established structurally (Section 4) and biochemically (Section 5.3). In *A. platensis* exposed to n-TiO_2_, toxicity was manifested through reduction in photosynthetic pigments and disappearance of phycobiliproteins, indicating that oxidative damage to photosynthetic components can precede and occur independently of bulk lipid peroxidation [15], and collectively these findings illustrate that n-TiO_2_-induced metabolomic transitions are concentration-dependent and disproportionately target the photosynthetic apparatus as a primary site of metabolic disruption.

### 8.4. Integrative Multi-Omics Approaches

The integration of transcriptomic, proteomic, and metabolomic data within systems biology frameworks offers the most comprehensive mechanistic account of cyanobacterial responses to n-TiO_2_ NPs and represents the frontier of understanding in this field. Multi-omics integration in cyanobacteria, encompassing transcriptomics, proteomics, metabolomics, and fluxomics, has established that effective deployment of these organisms as photosynthetic cell factories requires integrative metabolic modeling to identify rate-limiting steps and improve photosynthetic efficiency and bioproduct yields [43]. Applying these integrative frameworks to n-TiO_2_-exposed cyanobacterial systems holds promise for identifying master regulatory nodes whose modulation could amplify the beneficial photosynthetic enhancement effects of nanoparticles while suppressing toxicity pathways, and for building predictive metabolic models that guide rational optimization of n-TiO_2_ supplementation strategies in engineered bioproduction systems.

## 9. Artificial Intelligence-Guided Optimization of TiO_2_-Cyanobacterial Photosynthetic Systems

The integration of artificial intelligence into the optimization of n-TiO_2_-cyanobacterial photosynthetic systems represents a transformative methodological advancement that addresses the fundamental limitations of conventional iterative experimental screening. AI-driven computational frameworks, particularly protein structure prediction platforms such as AlphaFold2 and AlphaFold3, have achieved near-experimental accuracy in modeling three-dimensional protein conformations [82,83,84] (Table 4). These advances have enabled accurate structural prediction of key cyanobacterial photosynthetic targets, including ATP synthase subunits, PSII reaction center proteins such as the D1 subunit, and phycobiliprotein light-harvesting complexes. The AlphaFold Protein Structure Database, archiving predicted structures for over 214 million protein sequences, provides directly accessible structural inputs for modeling n-TiO_2_ interactions with cyanobacterial photosynthetic machinery [85]. Molecular docking platforms, including AutoDock Vina 1.2.0 and SwissDock 2024, extend these structural models into actionable screening workflows by estimating binding free energies and predicting ligand poses within ATP synthase and PSII core proteins, identifying molecular candidates capable of stabilizing photosynthetic complexes under n-TiO_2_ exposure or amplifying TiO_2_-mediated bioenergetic benefits [86,87] (Table 4).

At the systems level, machine learning models provide powerful predictive tools for resolving the concentration-dependent biological outcomes of n-TiO_2_ exposure in cyanobacterial systems. Quantitative structure–activity relationship models built on ensemble machine learning algorithms have been applied to predict n-TiO_2_ cytotoxicity outcomes, identifying particle size, surface area, zeta potential, and dosage as the dominant physicochemical determinants of biological response [88] (Table 4). Machine learning frameworks applied to nanomaterial-induced ROS dynamics have revealed the physicochemical parameters governing the critical transition between productive ROS-mediated adaptive signaling and oxidative cytotoxicity, and have demonstrated capacity to predict cytotoxic outcomes from nanoparticle descriptors prior to experimental validation, establishing a directly transferable framework for optimizing n-TiO_2_ exposure conditions in cyanobacterial photosynthetic systems [89]. AI-driven enzyme engineering platforms integrating protein structure prediction, generative sequence modeling, and molecular docking have demonstrated the capacity to accelerate rational mutagenesis, optimize catalytic function, and design novel biosynthetic enzymes with enhanced stability and activity, establishing a directly transferable framework for AI-guided enhancement of the cyanobacterial photosynthetic apparatus under n-TiO_2_ supplementation [90].

Complementing AI-driven approaches, statistical modeling frameworks provide a rigorous quantitative methodology for determining the optimal n-TiO_2_ concentrations that maximize cyanobacterial photosynthetic productivity. Multiple regression analysis coupled with design of experiments enables simultaneous quantification of the independent and interactive effects of n-TiO_2_ concentration, light intensity, and culture conditions on key photosynthetic output variables including biomass yield, chlorophyll content, and PSII quantum yield, providing a mathematically grounded basis for identifying dominant explanatory factors and characterizing their combined influence on photosynthetic performance [91]. Building on this foundation, nonlinear dose–response curve fitting applying four-parameter logistic and polynomial regression models is particularly well-suited to characterizing the biphasic concentration–response relationship consistently observed across cyanobacterial systems, enabling precise determination of EC_50_ thresholds that demarcate productive photosynthetic enhancement from oxidative toxicity [92,93]. Together, these statistical approaches translate empirical concentration–response data into quantitatively grounded supplementation thresholds that can be directly integrated into photobioreactor design protocols. The convergence of AI-guided structural biology, machine learning-based nanoparticle optimization, and statistical dose–response modeling therefore collectively establishes a rigorous, multiscale analytical framework that bridges molecular-level mechanistic understanding with scalable, reproducible, and biotechnologically deployable n-TiO_2_ supplementation strategies for enhanced cyanobacterial photosynthetic performance.

**Table 4:** AI and Computational Tools for Optimizing Titanium dioxide nanoparticle (n-TiO_2_)–Cyanobacterial Photosynthetic Systems.

Tool/Platform	Tool Type	Primary Function	Proposed Application to n-TiO_2_-Photosynthesis Optimization	Reference
Protein structure prediction				
AlphaFold2	Deep learning	Predicts 3D protein structures from amino acid sequences	Models cyanobacterial PSII D1 subunit, ATP synthase subunits, and phycobiliprotein complexes to identify n-TiO_2_ interaction sites	[82]
AlphaFold3	Diffusion-based deep learning	Extends structure prediction to protein–ligand, protein–nucleic acid, and multi-chain complexes	Enables modeling of n-TiO_2_ surface interactions with photosynthetic protein complexes; supports multi-subunit ATP synthase and PSII assembly modeling	[81]
AlphaFold Protein Structure Database	Structural database	Archives predicted structures for >214 million protein sequences	Provides accessible structural models of cyanobacterial photosynthetic proteins as inputs for molecular docking and nanoparticle interaction studies	[85]
Molecular docking				
AutoDock Vina 1.2.0	Stochastic docking algorithm	Estimates binding free energies and predicts ligand binding poses within defined protein regions	Screens small molecules targeting cyanobacterial ATP synthase β-subunit and PSII D1 protein to identify stabilizing ligands under n-TiO_2_ exposure	[87]
SwissDock 2024	Web-based docking platform	Integrates AutoDock Vina with Attracting Cavities algorithm for high-throughput small-molecule docking	Enables in silico screening of compounds that modulate photosynthetic enzyme activity or amplify n-TiO_2_ bioenergetic benefits in cyanobacteria	[86]
Machine learning and predictive modeling				
QSAR models (RF, GBM, XGBoost)	Ensemble machine learning	Predicts cytotoxicity of TiO_2_-based nanoparticles from physicochemical descriptors	Identifies particle size, surface area, zeta potential, and dosage as dominant determinants of n-TiO_2_ biological response; guides safe concentration selection for photosynthetic enhancement	[88]
ML-ROS modeling framework	Machine learning	Models ROS dynamics induced by nanomaterials including n-TiO_2_ from physicochemical parameters	Predicts the transition between ROS-mediated adaptive signaling and oxidative cytotoxicity; enables optimization of n-TiO_2_ exposure conditions for photosynthetic systems	[89]
Multi-omics and systems biology				
Multi-omic machine learning + GSMM	Integrative systems biology	Combines multi-omic datasets with genome-scale metabolic models using regularized flux balance analysis	Reveals mechanisms by which cyanobacteria cope with environmental fluctuations; provides a framework for modeling n-TiO_2_-induced metabolic reprogramming in photosynthetic systems	[94]
Transcriptomics/proteomics/metabolomics integration	Multi-omics pipeline	Applies transcriptomics, proteomics, metabolomics, and fluxomics to map cyanobacterial metabolism	Identifies master regulatory nodes governing n-TiO_2_ photosynthetic enhancement; informs predictive metabolic models for nanoparticle supplementation optimization	[83]
AI-guided enzyme and biosystem engineering				
AI enzyme engineering platform	Integrated AI platform	Integrates protein structure prediction, molecular docking, and spatial structure–activity relationship analysis to optimize multi-enzyme biosynthetic systems	Establishes precedent for AI-guided optimization of cyanobacterial photosynthetic apparatus; applicable to enhancing ATP synthase and PSII catalytic efficiency under n-TiO_2_ conditions	[90]

## 10. Challenges and Future Perspectives

Despite compelling laboratory evidence for n-TiO_2_-mediated enhancement of cyanobacterial photosynthesis, translating these findings into scalable biotechnological systems requires resolving several interconnected challenges spanning reproducibility, environmental risk, engineering feasibility, and techno-economic viability. Most existing studies have been conducted under idealized monoculture conditions with narrow concentration ranges and laboratory-scale culture volumes that bear limited resemblance to the optical, hydrodynamic, and chemical complexity of industrial photobioreactor environments. Addressing these challenges through coordinated experimental and computational approaches represents the critical next step toward realizing the full biotechnological potential of photocatalytic–biological coupling.

Among the challenges requiring immediate attention, the environmental fate and ecotoxicological consequences of n-TiO_2_ release into aquatic systems represent the most pressing concern for responsible deployment. Engineered n-TiO_2_ has been detected in municipal wastewater treatment plant effluents at concentrations ranging from less than 5 to 25 µg L^−1^, with influent levels reaching up to 1233 µg L^−1^ despite removal efficiencies exceeding 96% across multiple full-scale facilities [95,96]; sediment represents the dominant long-term environmental reservoir, with modeled freshwater concentrations reaching up to 25 mg kg^−1^, a compartmental partitioning that existing aquatic risk frameworks inadequately address [97,98]. Most ecotoxicological studies have focused on milligram-per-liter acute toxicity thresholds, failing to characterize sublethal effects on primary producer photosynthetic gene expression and microbial community composition that occur at environmentally relevant microgram-per-liter concentrations [49,98]. Any biotechnological deployment of n-TiO_2_-supplemented photobioreactor systems must therefore incorporate lifecycle analysis of nanoparticle fate after process termination, including effluent treatment protocols capable of quantitative nanoparticle removal before discharge into receiving water bodies.

Compounding the environmental challenge, the reproducibility of reported photosynthetic enhancement effects remains compromised by variability in nanoparticle physicochemical characterization, culture conditions, light regimes, and strain-specific responses. Studies employing identical nanoparticle concentrations but differing in crystal phase, particle size, illumination spectrum, or culture medium ionic composition produce qualitatively divergent outcomes that cannot be meaningfully compared or synthesized. The characterization framework presented in Table 1 represents a minimum reporting standard whose systematic adoption would substantially improve cross-study interpretability, and standardized experimental protocols modeled on OECD ecotoxicological testing guidelines should be developed and endorsed as prerequisites for publication in this field [15,49].

Beyond the laboratory, scaling n-TiO_2_ supplementation to industrially relevant photobioreactor systems introduces engineering and economic challenges that are entirely absent from the current literature. Self-shading in dense cyanobacterial cultures creates steep light gradients that drive photoinhibition at the illuminated surface while simultaneously imposing photo limitation in the culture interior, and the light-scattering properties of suspended n-TiO_2_ offer a potentially significant mitigation strategy by redistributing incident photons more uniformly throughout the culture volume [99]. However, the ionic composition of scaled growth media, which typically differs from laboratory BG-11 formulations in pH, salinity, and dissolved organic carbon content, will alter nanoparticle colloidal stability and effective photocatalytic surface area in ways that require systematic characterization before bench-scale optimized concentrations can be reliably translated [35]. The illumination spectrum of outdoor or greenhouse photobioreactors differs substantially from the UV-inclusive illumination of laboratory studies, meaning the photocatalytic contribution of unmodified n-TiO_2_ may be attenuated unless visible-light-active surface-modified formulations are specifically employed. Efficient recovery and reuse of n-TiO_2_ following cultivation is a further prerequisite for biotechnological viability, since particle loss both raises operating costs and increases the risk of environmental release. Membrane filtration, centrifugation, sedimentation, and magnetic functionalization of n-TiO_2_ have each been investigated as recovery strategies capable of preserving photocatalytic and biological activity across cultivation cycles [100], though their performance at production scale and their compatibility with continuous cyanobacterial cultivation remain to be established. Beyond these engineering considerations, nanoparticle material costs, feasibility of recovery and reuse across production cycles, risk of Ti contamination of harvested bioproducts, and requirements for effluent nanoparticle removal collectively demand rigorous techno-economic assessment and multidisciplinary collaboration between photobiologists, chemical engineers, and environmental scientists before deployment at scale can be responsibly pursued.

Future research should be directed toward resolving these challenges through integrated experimental and computational approaches. Multi-omics characterization of cyanobacterial responses to n-TiO_2_ across concentration gradients and illumination conditions is essential for identifying the master regulatory nodes governing the transition between productive photosynthetic enhancement and oxidative toxicity [83]. AI-guided identification of n-TiO_2_ surface modifications that maximize photosynthetic enhancement while minimizing cytotoxicity represents a particularly promising avenue, supported by the demonstrated capacity of machine learning frameworks to predict nanomaterial-induced ROS dynamics from physicochemical descriptors [88]. Molecular docking studies targeting ATP synthase subunits and PSII core proteins, enabled by AlphaFold-predicted structural models, should be prioritized to identify photosynthesis-stabilizing ligands amenable to nanoparticle surface conjugation [82,87]. Field-scale evaluation of cyanobacterial cultivation supplemented with optimized n-TiO_2_ concentrations, informed by nonlinear dose–response modeling and accompanied by rigorous environmental fate characterization, would provide the translational evidence base necessary to advance nano-enabled photosynthetic systems toward scalable and environmentally responsible biotechnological deployment [92].

## 11. Conclusions

Photocatalytic–biological coupling provides a mechanistically grounded framework for understanding how n-TiO_2_ enhances cyanobacterial photosynthesis at the interface of photophysics and cell biology. By functioning as artificial light amplifiers and ROS-calibrated redox modulators, n-TiO_2_ augment photon capture, accelerate electron transport, strengthen proton motive force, and elevate ATP synthase-driven energy output, collectively improving light utilization efficiency, pigment accumulation, and metabolic productivity across diverse cyanobacterial systems. These enhancements are concentration-dependent, strain-specific, and mediated by a dynamic nano-bio interface governed by nanoparticle physicochemical properties, cellular redox state, and the biochemical composition of the biological environment and reported enhancement or inhibition outcomes therefore cannot be extrapolated across studies without matching these parameters. Critically, the transition between productive photosynthetic enhancement and oxidative cytotoxicity is governed by a narrow and biologically defined ROS window whose precise boundaries vary across species, cultivation conditions, and nanoparticle formulations, underscoring the necessity of rigorous dose–response characterization as a prerequisite for any translational application. The integration of AI-guided structural biology, multi-omics systems analysis, and advanced nanomaterial engineering will be essential for resolving the mechanistic complexity of these interactions and for translating laboratory-scale findings into scalable, reproducible, and environmentally responsible biotechnological deployments in renewable energy production, carbon sequestration, and sustainable bioproduct synthesis.

## Figures and Tables

**Table 1:** Physicochemical characterization of n-TiO_2_ in cyanobacterial photosynthesis research.

Analytical Technique	Property Measured	Key Output Parameters	Biological Relevance in Cyanobacterial Systems	Key References
X-ray diffraction with Rietveld refinement	Crystal phase composition and crystallite size	Phase ratios (anatase, rutile, brookite); crystallite size (nm); lattice parameters	Determines electron–hole recombination rate and ROS yield; anatase phase confers superior photocatalytic activity relevant to PSII enhancement	[27,29,32]
X-ray photoelectron spectroscopy	Surface elemental composition and oxidation states	Ti^4+^/Ti^3+^ ratio; surface oxygen species; binding energies (eV)	Identifies surface defects and oxygen vacancies that modulate charge carrier generation and photocatalytic electron transfer to photosynthetic acceptors	[27,28]
Transmission and scanning electron microscopy	Primary particle size, morphology, and crystal facet distribution	Particle dimensions (nm); shape (spherical, rod, tube, sheet); exposed crystal facets	Governs surface-to-volume ratio and probability of interaction with cyanobacterial cell surfaces and thylakoid membranes; anatase {001} facets demonstrate higher photocatalytic reactivity than {101} facets	[12,34]
Dynamic light scattering and electrophoretic light scattering	Hydrodynamic diameter and colloidal stability in biological media	Hydrodynamic diameter (nm); polydispersity index (PDI); zeta potential (mV)	Quantifies agglomeration state in cyanobacterial culture media; effective hydrodynamic size determines actual biological dose and differs substantially from primary particle size under ionic culture conditions	[35]
N_2_ physisorption	Specific surface area and pore characteristics	Specific surface area (m^2^ g^−1^); pore volume (cm^3^ g^−1^); pore size distribution (nm)	Determines density of photocatalytically active sites and surface hydroxyl groups available for ROS generation; governs biomolecular corona formation at the nano–bio interface	[12,36]
Diffuse reflectance UV–visible spectroscopy with Tauc plot analysis	Optical bandgap and light absorption range	Bandgap energy (eV); absorption edge wavelength (nm); sub-bandgap absorption features	Determines spectral compatibility between n-TiO_2_ photocatalytic activation and photobioreactor illumination; pristine anatase absorbs below ~385 nm, requiring surface modification for visible-light activity in cyanobacterial cultures	[37,38]
Thermogravimetric analysis and diffuse reflectance infrared Fourier transform spectroscopy	Surface hydroxyl group density and composition	Ti-OH group concentration (mmol g^−1^); dehydration temperature ranges (°C); hydroxyl speciation	Predicts ROS speciation profile (·OH, O_2_^−^, H_2_O_2_) under defined illumination conditions; hydroxyl radical generation directly damages PSII reaction center proteins and membrane lipids proximal to the nanoparticle surface	[12,28]

**Table 2:** Concentration-dependent photosynthetic enhancement and inhibition thresholds of n-TiO_2_ across cyanobacteria and microalgae.

Organism	n-TiO_2_ Concentration Range Tested	Enhancement Threshold	Inhibition Threshold	Key Endpoints Measured	Crystal Phase/Particle Size	Reference
*F. diplosiphon* strains B481-SD (sterol desaturase overexpressor) and B481-WT (wild type)	0.5, 2.0, 4.0, 8.0, 16.0 mg L^−1^	B481-SD: 2.0 mg L^−1^ optimal; maximal growth (OD 0.67 ± 0.01); enhanced phycocyanin (1300 ± 2) and chlorophyll a (890 ± 5); increased PSII efficiency and ATP synthase activity	B481-WT: reduced PSII response and no significant pigment changes across all concentrations; strain-specific differential sensitivity demonstrated	Growth (OD), phycocyanin, chlorophyll a autofluorescence, PSII quantum yield (Fv/Fm), ROS, ATP synthase activity	Anatase; ~21 nm	[46]
*Synechocystis* sp. PCC 6803	25 mg L^−1^	Significant enhancement: chlorophyll content +62.2% (14.6 vs. 9.0 mg L^−1^ in control); phycocyanin +33.8%; allophycocyanin +55.0%; thylakoid membrane lipid remodeling observed	Growth characteristics not significantly affected at tested concentration	Chlorophyll content, phycocyanin, allophycocyanin, thylakoid lipid composition (DI-MS)	Anatase; <25 nm	[47]
*Synechocystis* sp. PCC 6803	10 mg L^−1^	petF (ferredoxin) upregulated; EPS secretion increased as protective response	10 mg L^−1^: significant PSII inhibition; psb28-2 downregulated; ROS overproduction; disruption of photosynthetic protein complexes and electron transport; growth inhibition	Transcriptomics (RNA-seq), PSII quantum yield, ROS levels, growth rate, EPS production	Anatase P25; 10 and 50 nm	[48]
*Arthrospira platensis*	0.01 mg L^−1^–30 mg L^−1^	Low concentrations: enhanced PSII activity, improved total protein and pigment yields; ATP content elevation	High concentrations: reduction in photosynthetic pigments, disappearance of phycobiliproteins, lipid peroxidation (MDA accumulation), reduced cell viability	Chlorophyll a, phycocyanin, allophycocyanin, PSII activity, MDA, ATP content, protein yield, ROS	Anatase; ~21 nm	[15]
*A. platensis* UTEX1926	100 mg L^−1^	N-doped anatase: increased extracellular polyphenol production (68.0 mg g^−1^ DB) indicating antioxidant adaptation	Pure anatase: 74.1% growth inhibition; 15% N-doped anatase: significant polyphenol stress response	Biomass concentration, polyphenol content (intracellular and extracellular), cell morphology	Pure anatase:14.02 ± 15% N-doped anatase: 17.13 ± 2.00 nm	[45]
*Chlorella vulgaris*	1.1, 4.4, 8.8, 17.6 mg L^−1^	8.8 to 17.6 µg L^−1^: upregulation of psaA, psaB, psaD (PSI), psbA (D1), atpB (ATP synthase β-subunit); hormetic growth stimulation at intermediate concentrations	1.1 to 4.4 µg L^−1^: significant rbcL repression indicating sublethal CO_2_ fixation impairment not detectable by growth measurements	Transcriptomics (psaA, psaB, psaD, psbA, rbcL, atpB), growth rate, PSII quantum yield	Anatase dominant; <100 nm primary particles	[49]
*Microcystis aeruginosa*	0.5, 2.5, 5, 10, 25 mg L^−1^	No significant enhancement reported; *Synechococcus cedrorum* showed metabolic activity increase at low doses	IC_25_ = 2.7 mg L^−1^ (60 min); metabolic activity never fell below 40% of control at 25 mg L^−1^; substantially less sensitive than microalgal species (6–10-fold higher IC_25_)	Metabolic activity, cell viability	P25 (anatase-rutile mixed phase); 81.5 nm crystallite size	[50]

**Table 3:** Photosynthesis-Related Gene Responses to Titanium dioxide nanoparticles in Cyanobacteria and Microalgae.

Gene	Encoded Protein/Function	Organism	n-TiO_2_ Concentration	Expression Response	Reference
Photosystem I (PSI)					
psaA	PSI reaction center protein A	*Chlorella vulgaris*	8.8 to 17.6 µg L^−1^	Upregulated	[49]
psaB	PSI reaction center protein B	*Chlorella vulgaris*	8.8 to 17.6 µg L^−1^	Upregulated	[49]
psaD	PSI subunit II; ferredoxin docking	*Chlorella vulgaris*	8.8 to 17.6 µg L^−1^	Upregulated	[49]
psaI	PSI subunit VIII; stabilizes PSI trimer	*Chlorella pyrenoidosa*	15 mg L^−1^	Upregulated	[63]
Photosystem II (PSII)					
psbA	D1 reaction center protein; water oxidation	*Chlorella vulgaris*	8.8 to 17.6 µg L^−1^	Upregulated	[49]
psbA	D1 reaction center protein; water oxidation	*Chlorella pyrenoidosa*	15 mg L^−1^	Upregulated	[63]
psbM	PSII reaction center protein M; structural subunit	*Chlorella pyrenoidosa*	15 mg L^−1^	Upregulated	[63]
psbK	PSII reaction center protein K; structural subunit	*Chlorella pyrenoidosa*	15 mg L^−1^	Upregulated	[63]
psb28-2	PSII assembly and repair protein	*Synechocystis* sp. PCC 6803	10 µg L^−1^	Downregulated	[48]
Carbon fixation					
rbcL	RuBisCO large subunit; CO_2_ fixation	*Chlorella vulgaris*	1.1 to 4.4 µg L^−1^	Downregulated	[49]
rbcL	RuBisCO large subunit; CO_2_ fixation	*Chlorella vulgaris*	8.8 to 17.6 µg L^−1^	Upregulated	[49]
ATP synthesis					
atpB	ATP synthase β-subunit; F_1_ catalytic head	*Chlorella vulgaris*	8.8 to 17.6 µg L^−1^	Upregulated	[49]
Electron transport and PSII assembly					
petF	Ferredoxin; photosynthetic electron transfer from PSI	*Synechocystis* sp. PCC 6803	10 µg L^−1^	Upregulated	[48]
Oxidative stress and antioxidant response					
sodB	Fe-superoxide dismutase; ROS detoxification	*Synechocystis* sp. PCC 6803	10 µg L^−1^	Upregulated	[48]
katG	Catalase-peroxidase; H_2_O_2_ degradation	*Synechocystis* sp. PCC 6803	10 µg L^−1^	Upregulated	[48]

## Data Availability

All information has been included in the manuscript.

## References

[1] Sánchez-Baracaldo P., Cardona T. (2020). On the origin of oxygenic photosynthesis and Cyanobacteria. New Phytol..

[2] Agarwal P., Soni R., Kaur P., Madan A., Mishra R., Pandey J., Singh S., Singh G. (2022). Cyanobacteria as a promising alternative for sustainable environment: Synthesis of biofuel and biodegradable plastics. Front. Microbiol..

[3] Pagels F., Guedes A.C., Amaro H.M., Kijjoa A., Vasconcelos V. (2019). Phycobiliproteins from cyanobacteria: Chemistry and biotechnological applications. Biotechnol. Adv..

[4] Santos-Merino M., Torrado A., Davis G.A., Röttig A., Bibby T.S., Kramer D.M., Ducat D.C. (2021). Improved photosynthetic capacity and photosystem I oxidation via heterologous metabolism engineering in cyanobacteria. Proc. Natl. Acad. Sci. USA.

[5] Melis A. (2009). Solar energy conversion efficiencies in photosynthesis: Minimizing the chlorophyll antennae to maximize efficiency. Plant Sci..

[6] Nikkanen L., Solymosi D., Jokel M., Allahverdiyeva Y. (2021). Regulatory electron transport pathways of photosynthesis in cyanobacteria and microalgae: Recent advances and biotechnological prospects. Physiol. Plant..

[7] Stadnichuk I.N., Kusnetsov V.V. (2023). Phycobilisomes and phycobiliproteins in the pigment apparatus of oxygenic photosynthetics: From cyanobacteria to tertiary endosymbiosis. Int. J. Mol. Sci..

[8] Luimstra V.M., Schuurmans J.M., Verschoor A.M., Hellingwerf K.J., Huisman J., Matthijs H.C.P. (2018). Blue light reduces photosynthetic efficiency of cyanobacteria through an imbalance between photosystems I and II. Photosynth. Res..

[9] Wyatt L., Gichuki S., Yalcin Y.S., Sitther V. (2023). Impact of ascorbic acid on zero-valent iron nanoparticle and UV-B mediated stress in the cyanobacterium, *Fremyella diplosiphon*. Microorganisms.

[10] Mullineaux C.W. (2014). Co-existence of photosynthetic and respiratory activities in cyanobacterial thylakoid membranes. Biochim. Biophys. Acta (BBA)—Bioenerg..

[11] Rathore C., Yadav V.K., Gacem A., AbdelRahim S.K., Verma R.K., Chundawat R.S., Gnanamoorthy G., Yadav K.K., Choudhary N., Sahoo D.K. (2023). Microbial synthesis of titanium dioxide nanoparticles and their importance in wastewater treatment and antimicrobial activities: A review. Front. Microbiol..

[12] Wang Q., Li Y., Huang F., Song S., Ai G., Xin X., Zhao B., Zheng Y., Zhang Z. (2023). Recent advances in g-C_3_N_4_-based materials and their application in energy and environmental sustainability. Molecules.

[13] Shivaji K., Sridharan K., Kirubakaran D.D., Velusamy J., Emadian S.S., Krishnamurthy S., Devadoss A., Nagarajan S., Das S., Pitchaimuthu S. (2023). Biofunctionalized CdS quantum dots: A case study on nanomaterial toxicity in the photocatalytic wastewater treatment process. ACS Omega.

[14] Li P., Xia Y., Song K., Liu D. (2024). The impact of nanomaterials on photosynthesis and antioxidant mechanisms in gramineae plants: Research progress and future prospects. Plants.

[15] Rudi L., Cepoi L., Chiriac T., Djur S. (2025). Interactions between potentially toxic nanoparticles (Cu, CuO, ZnO, and TiO_2_) and the cyanobacterium *Arthrospira platensis*: Biological adaptations to xenobiotics. Nanomaterials.

[16] Grettenberger C.L., Abou-Shanab R., Hamilton T.L. (2024). Limiting factors in the operation of photosystems I and II in cyanobacteria. Microb. Biotechnol..

[17] Nishiyama Y., Allakhverdiev S.I., Murata N. (2011). Protein synthesis is the primary target of reactive oxygen species in the photoinhibition of photosystem II. Physiol. Plant..

[18] Muzzopappa F., Kirilovsky D. (2020). Changing color for photoprotection: The orange carotenoid protein. Trends Plant Sci..

[19] Shimakawa G. (2023). Electron transport in cyanobacterial thylakoid membranes: Are cyanobacteria simple models for photosynthetic organisms?. J. Exp. Bot..

[20] Murata N., Takahashi S., Nishiyama Y., Allakhverdiev S.I. (2007). Photoinhibition of photosystem II under environmental stress. Biochim. Biophys. Acta (BBA)—Bioenerg..

[21] Singh V.K., Jha S., Rana P., Mishra S., Kumari N., Singh S.C., Anand S., Upadhye V., Sinha R.P. (2023). Resilience and mitigation strategies of cyanobacteria under ultraviolet radiation stress. Int. J. Mol. Sci..

[22] Zhang J., Lee K.P., Liu Y., Kim C. (2025). Temperature-driven changes in membrane fluidity differentially impact FILAMENTATION TEMPERATURE-SENSITIVE H2-mediated photosystem II repair. Plant Cell.

[23] Li Y., Wang H., Tang L., Zhu H. (2023). Titanium dioxide nanoparticles enhance photocurrent generation of cyanobacteria. Biochem. Biophys. Res. Commun..

[24] Schneider J., Matsuoka M., Takeuchi M., Zhang J., Horiuchi Y., Anpo M., Bahnemann D.W. (2014). Understanding TiO_2_ photocatalysis: Mechanisms and materials. Chem. Rev..

[25] Lettieri S., Pavone M., Fioravanti A., Santamaria Amato L., Maddalena P. (2021). Charge carrier processes and optical properties in TiO_2_ and TiO_2_-based heterojunction photocatalysts: A review. Materials.

[26] Qian R., Zong H., Schneider J., Zhou G., Zhao T., Li Y., Yang J., Bahnemann D.W., Pan J.H. (2019). Charge carrier trapping, recombination and transfer during TiO_2_ photocatalysis: An overview. Catal. Today.

[27] Luttrell T., Halpegamage S., Tao J., Kramer A., Sutter E., Batzill M. (2014). Why is anatase a better photocatalyst than rutile? Model studies on epitaxial TiO_2_ films. Sci. Rep..

[28] Nosaka Y., Nosaka A.Y. (2017). Generation and detection of reactive oxygen species in photocatalysis. Chem. Rev..

[29] Hurum D.C., Agrios A.G., Gray K.A., Rajh T., Thurnauer M.C. (2003). Explaining the enhanced photocatalytic activity of Degussa P25 mixed-phase TiO_2_ using EPR. J. Phys. Chem. B.

[30] Ohtani B., Prieto-Mahaney O.O., Li D., Abe R. (2010). What is Degussa (Evonik) P25? Crystalline composition analysis, reconstruction from isolated pure particles and photocatalytic activity test. J. Photochem. Photobiol. A Chem..

[31] Vequizo J.J.M., Matsunaga H., Ishiku T., Kamimura S., Ohno T., Yamakata A. (2017). Trapping-induced enhancement of photocatalytic activity on brookite TiO_2_ powders: Comparison with anatase and rutile TiO_2_ powders. ACS Catal..

[32] Yamakata A., Vequizo J.J.M., Matsunaga H. (2015). Distinctive behavior of photogenerated electrons and holes in anatase and rutile TiO_2_ powders. J. Phys. Chem. C.

[33] Yang H.G., Sun C.H., Qiao S.Z., Zou J., Liu G., Smith S.C., Cheng H.M., Lu G.Q. (2008). Anatase TiO_2_ single crystals with a large percentage of reactive facets. Nature.

[34] Liu S., Yu J., Jaroniec M. (2011). Anatase TiO_2_ with dominant high-energy {001} facets: Synthesis, properties, and applications. Chem. Mater..

[35] Gonzalo S., Llaneza V., Pulido-Reyes G., Fernández-Piñas F., Bonzongo J.C., Leganes F., Rosal R., García-Calvo E., Rodea-Palomares I. (2014). A colloidal singularity reveals the crucial role of colloidal stability for nanomaterials in vitro toxicity testing: nZVI-microalgae colloidal system as a case study. PLoS ONE.

[36] Du T., Meng R., Qian L., Wang Z., Li T., Wu L. (2024). Formation of extracellular polymeric substances corona on TiO_2_ nanoparticles: Roles of crystalline phase and exposed facets. Water Res..

[37] Ghumro S.S., Lal B., Pirzada T. (2022). Visible-light-driven carbon-doped TiO_2_-based nanocatalysts for enhanced activity toward microbes and removal of dye. ACS Omega.

[38] Fujishima A., Zhang X., Tryk D.A. (2008). TiO_2_ photocatalysis and related surface phenomena. Surf. Sci. Rep..

[39] Etacheri V., Di Valentin C., Schneider J., Bahnemann D., Pillai S.C. (2015). Visible-light activation of TiO_2_ photocatalysts: Advances in theory and experiments. J. Photochem. Photobiol. C Photochem. Rev..

[40] Glemser M., Heining M., Schmidt J., Becker A., Garbe D., Buchholz R., Brück T. (2016). Application of light-emitting diodes (LEDs) in cultivation of phototrophic microalgae: Current state and perspectives. Appl. Microbiol. Biotechnol..

[41] Miller R.J., Bennett S., Keller A.A., Pease S., Lenihan H.S. (2012). TiO_2_ nanoparticles are phototoxic to marine phytoplankton. PLoS ONE.

[42] Liu Q., Li X., Chen Y., Zhang X., Gao B., Ma M., Yang H., Yuan S., Zhang Q. (2025). Strategies for Regulating Reactive Oxygen Species in Carbon Nitride-Based Photocatalysis. Molecules.

[43] Asahi R., Morikawa T., Ohwaki T., Aoki K., Taga Y. (2001). Visible-light photocatalysis in nitrogen-doped titanium oxides. Science.

[44] Choi H., Antoniou M.G., Pelaez M., de la Cruz A.A., Shoemaker J.A., Dionysiou D.D. (2007). Mesoporous nitrogen-doped TiO_2_ for the photocatalytic destruction of the cyanobacterial toxin microcystin-LR under visible light irradiation. Environ. Sci. Technol..

[45] Comotto M., Casazza A.A., Aliakbarian B., Caratto V., Ferretti M., Perego P. (2014). Influence of TiO_2_ nanoparticles on growth and phenolic compounds production in photosynthetic microorganisms. Sci. World J..

[46] Sayadujjhara M., Yalcin Y.S., Ghann W., Uddin J., Sitther V. (2025). Strain-specific impact of titanium dioxide nanoparticles on *Fremyella diplosiphon* physiological and metabolic responses. Curr. Microbiol..

[47] Zahra Z., Kim S.Y., Kim H.Y., Lee H., Lee H., Jeon J.Y., Kim D.-M., Kim D.-M., Hong S.-J., Cho B.-K. (2018). Phycobiliproteins production enhancement and lipidomic alteration by titanium dioxide nanoparticles in *Synechocystis* sp. PCC 6803 culture. J. Agric. Food Chem..

[48] Xu K., Li Z., Juneau P., Xiao F., Lian Y., Zhang W., Shu L., Jiang H., Zhang K., Wang C. (2021). Toxic and protective mechanisms of cyanobacterium *Synechocystis* sp. in response to titanium dioxide nanoparticles. Environ. Pollut..

[49] Gutiérrez G.G., Rivas-Valdés F., Benavente B.P., Olivares R., Hepp M.I., Barra R.O., Urrutia R. (2025). Toxicological responses of photosynthetic genes in *Chlorella vulgaris* exposed to environmentally relevant concentrations of TiO_2_ nanoparticles. Int. J. Mol. Sci..

[50] Binh C.T.T., Peterson C.G., Tong T., Gray K.A., Gaillard J.F., Kelly J.J. (2015). Comparing acute effects of a nano-TiO_2_ pigment on cosmopolitan freshwater phototrophic microbes using high-throughput screening. PLoS ONE.

[51] Makuła P., Pacia M., Macyk W. (2018). How to correctly determine the band gap energy of modified semiconductor photocatalysts based on UV–Vis spectra. J. Phys. Chem. Lett..

[52] Latifi A., Ruiz M., Zhang C.C. (2009). Oxidative stress in cyanobacteria. FEMS Microbiol. Rev..

[53] Khedr T.M., El-Sheikh S.M., Ismail A.A., Kowalska E., Bahnemann D.W. (2019). Photodegradation of Microcystin-LR Using Visible Light-Activated C/N-co-Modified Mesoporous TiO_2_ Photocatalyst. Materials.

[54] Sies H., Jones D.P. (2020). Reactive oxygen species (ROS) as pleiotropic physiological signalling agents. Nat. Rev. Mol. Cell Biol..

[55] Kim W., Tachikawa T., Moon G.-h., Majima T., Choi W. (2014). Molecular-level understanding of the photocatalytic activity difference between anatase and rutile nanoparticles. Angew. Chem. Int. Ed..

[56] Hirakawa T., Yawata K., Nosaka Y. (2007). Photocatalytic reactivity for O_2_·^−^ and ·OH radical formation in anatase and rutile TiO_2_ suspension as the effect of H_2_O_2_ addition. Appl. Catal. A Gen..

[57] Mueller R., Kammler H.K., Wegner K., Pratsinis S.E. (2003). OH surface density of SiO_2_ and TiO_2_ by thermogravimetric analysis. Langmuir.

[58] Önal Acet B., Gül D., Stauber R.H., Odabaşı M., Acet Ö. (2024). A review for uncovering the “protein–nanoparticle alliance”: Implications of the protein corona for biomedical applications. Nanomaterials.

[59] Bilardo R., Traldi F., Vdovchenko A., Resmini M. (2022). Influence of surface chemistry and morphology of nanoparticles on protein corona formation. Wiley Interdiscip. Rev. Nanomed. Nanobiotechnol..

[60] Adeleye A.S., Keller A.A. (2016). Interactions between algal extracellular polymeric substances and commercial TiO_2_ nanoparticles in aqueous media. Environ. Sci. Technol..

[61] Pereira S., Zille A., Micheletti E., Moradas-Ferreira P., De Philippis R., Tamagnini P. (2009). Complexity of cyanobacterial exopolysaccharides: Composition, structures, inducing factors and putative genes involved in their biosynthesis and assembly. FEMS Microbiol. Rev..

[62] Cherchi C., Chernenko T., Diem M., Gu A.Z. (2011). Impact of nano titanium dioxide exposure on cellular structure of *Anabaena variabilis* and evidence of internalization. Environ. Toxicol. Chem..

[63] Middepogu A., Hou J., Gao X., Lin D. (2018). Effect and mechanism of TiO_2_ nanoparticles on the photosynthesis of *Chlorella pyrenoidosa*. Ecotoxicol. Environ. Saf..

[64] Liu L.N. (2016). Distribution and dynamics of electron transport complexes in cyanobacterial thylakoid membranes. Biochim. Biophys. Acta (BBA)—Bioenerg..

[65] Vatankhah A., Aliniaeifard S., Moosavi-Nezhad M., Abdi S., Mokhtarpour Z., Reezi S., Tsaniklidis G., Fanourakis D. (2023). Plants exposed to titanium dioxide nanoparticles acquired contrasting photosynthetic and morphological strategies depending on the growing light intensity: A case study in radish. Sci. Rep..

[66] Gao F., Hong F., Liu C., Zheng L., Su M., Wu X., Yang F., Wu C., Yang P. (2006). Mechanism of nano-anatase TiO_2_ on promoting photosynthetic carbon reaction of spinach. Biol. Trace Elem. Res..

[67] Lei Z., Mingyu S., Chao L., Liang C., Hao H., Xiao W., Xiaoqing L., Fan Y., Fengqing G., Fashui H. (2007). Effects of nano-anatase TiO_2_ on photosynthesis of spinach chloroplasts under different light illumination. Biol. Trace Elem. Res..

[68] Boden J.S., Konhauser K.O., Robbins L.J., Sánchez-Baracaldo P. (2021). Timing the evolution of antioxidant enzymes in cyanobacteria. Nat. Commun..

[69] Qiao Y., Wang W., Lu X. (2020). High light induced alka(e)ne biodegradation for lipid and redox homeostasis in cyanobacteria. Front. Microbiol..

[70] Zhang C., He Q., Zhan S., Zhou F. (2023). Photocatalytic hydrogen peroxide production in natural seawater by AgQDs(0D)/Bi_2_O_3_(3D) hybrid structure for in situ bacterial inactivation. J. Photochem. Photobiol. A Chem..

[71] Sies H., Berndt C., Jones D.P. (2017). Oxidative stress. Annu. Rev. Biochem..

[72] Yi S., Guo X., Lou W., Mao S., Luan G., Lu X. (2024). Structure, regulation, and significance of cyanobacterial and chloroplast adenosine triphosphate synthase in the adaptability of oxygenic photosynthetic organisms. Microorganisms.

[73] Junge W., Nelson N. (2015). ATP synthase. Annu. Rev. Biochem..

[74] Davis G.A., Kramer D.M. (2020). Optimization of ATP synthase c-rings for oxygenic photosynthesis. Front. Plant Sci..

[75] Song K., Baumgartner D., Hagemann M., Muro-Pastor A.M., Maaß S., Becher D., Hess W.R. (2022). AtpΘ is an inhibitor of F_0_F_1_ ATP synthase to arrest ATP hydrolysis during low-energy conditions in cyanobacteria. Curr. Biol..

[76] Shanmuganathan R., Le Q.H., Aloufi A.S., Gavurová B., Deepak J.R., Mosisa E., Praveenkumar T.R. (2023). High efficiency lipid production, biochar yield and chlorophyll a content of *Chlorella* sp. microalgae exposed to seawater and TiO_2_ nanoparticles. Environ. Res..

[77] Wang S., Alenius H., El-Nezami H., Karisola P. (2022). A new look at the effects of engineered ZnO and TiO_2_ nanoparticles: Evidence from transcriptomics studies. Nanomaterials.

[78] Yang M.K., Qiao Z.X., Zhang W.Y., Xiong Q., Zhang J., Li T., Ge F., Zhao J.-D. (2013). Global phosphoproteomic analysis reveals diverse functions of serine/threonine/tyrosine phosphorylation in the model cyanobacterium *Synechococcus* sp. strain PCC 7002. J. Proteome Res..

[79] Babele P.K., Kumar J., Chaturvedi V. (2019). Proteomic de-regulation in cyanobacteria in response to abiotic stresses. Front. Microbiol..

[80] Satti S.H., Raja N.I., Ikram M., Oraby H.F., Mashwani Z.U.R., Mohamed A.H., Singh A., Omar A.A. (2022). Plant-based titanium dioxide nanoparticles trigger biochemical and proteome modifications in *Triticum aestivum* L. under biotic stress of *Puccinia striiformis*. Molecules.

[81] Liu W., Li M., Li W., Keller A.A., Slaveykova V.I. (2022). Metabolic alterations in alga *Chlamydomonas reinhardtii* exposed to nTiO_2_ materials. Environ. Sci. Nano.

[82] Abramson J., Adler J., Dunger J., Evans R., Green T., Pritzel A., Ronneberger O., Willmore L., Ballard A.J., Bambrick J. (2024). Accurate structure prediction of biomolecular interactions with AlphaFold 3. Nature.

[83] Pathania R., Srivastava A., Srivastava S., Shukla P. (2022). Metabolic systems biology and multi-omics of cyanobacteria: Perspectives and future directions. Bioresour. Technol..

[84] Jumper J., Evans R., Pritzel A., Green T., Figurnov M., Ronneberger O., Tunyasuvunakool K., Bates R., Žídek A., Potapenko A. (2021). Highly accurate protein structure prediction with AlphaFold. Nature.

[85] Varadi M., Bertoni D., Magana P., Paramval U., Pidruchna I., Radhakrishnan M., Tsenkov M., Nair S., Mirdita M., Yeo J. (2024). AlphaFold Protein Structure Database in 2024: Providing structure coverage for over 214 million protein sequences. Nucleic Acids Res..

[86] Bugnon M., Röhrig U.F., Goullieux M., Perez M.A.S., Daina A., Michielin O., Zoete V. (2024). SwissDock 2024: Major enhancements for small-molecule docking with Attracting Cavities and AutoDock Vina. Nucleic Acids Res..

[87] Eberhardt J., Santos-Martins D., Tillack A.F., Forli S. (2021). AutoDock Vina 1.2.0: New docking methods, expanded force field, and Python bindings. J. Chem. Inf. Model..

[88] Roy J., Pore S., Roy K. (2023). Prediction of cytotoxicity of heavy metals adsorbed on nano-TiO_2_ with periodic table descriptors using machine learning approaches. Beilstein J. Nanotechnol..

[89] Ji Z., Yin Z. (2025). Machine learning-driven prediction of reactive oxygen species dynamics for assessing nanomaterials’ cytotoxicity. Biomimetics.

[90] Khan M.F., Khan M.T. (2026). AI-driven enzyme engineering: Emerging models and next-generation biotechnological applications. Molecules.

[91] Gérin S., Mathy G., Franck F. (2014). Modeling the dependence of respiration and photosynthesis upon light, acetate, carbon dioxide, nitrate and ammonium in *Chlamydomonas reinhardtii* using design of experiments and multiple regression. BMC Syst. Biol..

[92] Ritz C., Baty F., Streibig J.C., Gerhard D. (2015). Dose-response analysis using R. PLoS ONE.

[93] Ritz C., Streibig J.C. (2005). Bioassay analysis using R. J. Stat. Softw..

[94] Vijayakumar S., Rahman P.K.S.M., Angione C. (2020). A hybrid flux balance analysis and machine learning pipeline elucidates metabolic adaptation in cyanobacteria. iScience.

[95] Kiser M.A., Westerhoff P., Benn T., Wang Y., Pérez-Rivera J., Hristovski K. (2009). Titanium nanomaterial removal and release from wastewater treatment plants. Environ. Sci. Technol..

[96] Westerhoff P., Song G., Hristovski K., Kiser M.A. (2011). Occurrence and removal of titanium at full-scale wastewater treatment plants: Implications for TiO_2_ nanomaterials. J. Environ. Monit..

[97] Praetorius A., Scheringer M., Hungerbühler K. (2012). Development of environmental fate models for engineered nanoparticles—A case study of TiO_2_ nanoparticles in the Rhine River. Environ. Sci. Technol..

[98] Farkas J., Peter H., Ciesielski T.M., Thomas K.V., Sommaruga R., Salvenmoser W., Weyhenmeyer G.A., Tranvik L.J., Jenssen B.M. (2015). Impact of TiO_2_ nanoparticles on freshwater bacteria from three Swedish lakes. Sci. Total Environ..

[99] Saccardo A., Bezzo F., Sforza E. (2022). Microalgae growth in ultra-thin steady-state continuous photobioreactors: Assessing self-shading effects. Front. Bioeng. Biotechnol..

[100] Bockenstedt J., Vidwans N.A., Gentry T., Vaddiraju S. (2021). Catalyst recovery, regeneration and reuse during large-scale disinfection of water using photocatalysis. Water.

